# Dienogest-induced major depressive disorder with suicidal ideation: A case report

**DOI:** 10.1097/MD.0000000000027456

**Published:** 2021-10-08

**Authors:** Sang Min Lee, Jin Kyung Park

**Affiliations:** a Department of Psychiatry, Kyung Hee University Hospital, Seoul, Republic of Korea; b Department of Psychiatry, Kyung Hee University Hospital at Gangdong, Seoul, Republic of Korea.

**Keywords:** case report, dienogest, endometriosis, major depressive disorder, progestins, suicidal ideation

## Abstract

**Rationale:**

Dienogest is a type of progestin used for the treatment of endometriosis (EM). However, a significant adverse effect of dienogest is depression; therefore, assessing for a history of mood disorders is recommended before prescribing the drug. Herein, we present the case of a patient with no history of psychiatric disorders who was diagnosed with dienogest-induced major depressive disorder. This case emphasizes the importance of close monitoring for negative mood changes in patients taking dienogest.

**Patient concerns:**

A 41-year-old woman underwent surgery for EM. Postoperatively, her gynecologist prescribed dienogest (2 mg/d) to control EM symptoms. Two months after the initiation of dienogest, she manifested insomnia almost daily, gradually became depressed, lost interest in all activities, had incessant cries, and repeatedly thought of death. She had no history of major physical or psychiatric disorders.

**Diagnosis:**

Major depressive disorder, single episode, severe.

**Interventions:**

A psychiatric consultation was recommended, an antidepressant was prescribed, and dienogest was discontinued.

**Outcomes:**

Two weeks later, there was significant improvement in the symptoms, and after 4 weeks, she remained in a stable mood with no suicidal thoughts. She was followed up for 13 months with a maintenance dose of escitalopram (5 −10mg/d), until the psychiatrist recommended treatment discontinuation, with a confirmed state of remission.

**Lessons:**

This was a case of dienogest-induced depression in a patient with no history of mood disorders. Clinicians should be aware of the possibility of the occurrence of severe depression in progestin users regardless of their previous history.

## 1 Introduction

Dienogest belongs to a class of progestins, which are drugs that act like progesterone and are frequently prescribed for the treatment of endometriosis (EM). It has common adverse effects, such as metrorrhagia, constipation, headache, and hot flushes.^[1]^ Although depression is one of the significant adverse effects of dienogest, a clear association has not yet been established.^[2]^ However, it is recommended that clinicians should evaluate for a history of previous or ongoing mood disorders before prescribing dienogest. Depression of mild to moderate intensity associated with dienogest has been reported in most previous studies on the use of dienogest for the treatment of EM.^[3–5]^ We report a case of severe major depressive disorder (MDD) with suicidal thoughts induced by dienogest in a patient who had no history of mood disorders. This case emphasizes the need for close observation for mood fluctuations in patients on this therapy.

## 2 Case presentation

A 41-year-old woman visited the psychiatric department's outpatient clinic with complaints of depressed mood and insomnia. Six months before presentation, she had undergone left salpingo-oophorectomy for EM. Injection of leuprorelin acetate (3.75 mg per month) was administered for 2 months for the treatment of EM. Dienogest (2 mg/d) was prescribed for 3 months to control EM symptoms. She had difficulties in initiating sleep and stayed up at night almost daily for 2 months since starting dienogest. She remained depressed for most of the day, had lost interest in almost all activities of her life, got upset easily, cursed, cried for several hours, and repeatedly thought of death. This occurred for 2 weeks until she was referred to the psychiatric department by her gynecologist. On her visit to the psychiatric outpatient clinic, she complained of sleep problems and feelings of depression, lethargy, hopelessness, and anger. She had no pleasure or desire for daily life and had suicidal thoughts. She wept throughout the psychiatric interview and said that she was unable to control her weeping. She had no history of major physical diseases nor psychiatric disorders, except EM. She had never attempted suicide and has no family history of psychiatric disorders. Considering the progression of the mood symptoms related to medication and her personal history, the psychiatrist diagnosed this case as a treatment-related adverse event. On arrival at the psychiatric clinic, her blood pressure was 130/94 mmHg, and pulse rate was 128 bpm. Physical or neurological examinations were unremarkable. She was diagnosed by the psychiatrist with MDD, single episode, severe according to the Diagnostic and Statistical Manual of Mental Disorders, Fifth Edition.

The severity of her depression and anxiety was evaluated by the following scales. Her score on the Patient Health Questionnaire-9 (PHQ-9) was 16 (indicative of severe depression) against a normal score of 4; the Beck Depression Inventory (BDI) score was 43, which also indicated severe depression (the score for mild depression on this scale is 13), and the Beck Anxiety Inventory (BAI) score was 44 indicative of severe anxiety, against a normal score of 5. Desvenlafaxine (50 mg/d; for the depressed mood) and zolpidem (10 mg/d; for insomnia) were prescribed, and dienogest was discontinued. After 2 weeks, she showed significant improvement in the symptoms associated with MDD, including the depressed mood, anergia, and rage. She was able to fall asleep more easily and had a decreased frequency of crying spells and suicidal thoughts. Escitalopram (5 mg/d) was prescribed instead of desvenlafaxine due to high blood pressure detected during the visit, and zolpidem (10 mg/d) was prescribed again. At the 4-week follow-up, she was in a stable mood and could perform daily activities; however, some sleep problems persisted, although with no more suicidal thoughts. Escitalopram (5 mg/d) was continued, and at the 10-week follow-up since the first visit to the psychiatric outpatient clinic, the severity of her symptoms was evaluated again on following scales: the PHQ-9 score was 4, the BDI score was 13, and the BAI score was 5. She was advised to continue escitalopram for a few months, and the gynecologist suggested that dienogest therapy was no longer required. At the 14-week follow-up, the psychiatrist increased the dosage of escitalopram from 5 to 10 mg/d for the continued depression and anxiety, and it was maintained for 6 months. At 6 months, she was found to be in remission and stable, and the dose was tapered under close observation. At the 13-month visit, the medication was discontinued after the psychiatrist confirmed that the patient was in a stable state.

## 3 Discussion

EM is a chronic disease with a high rate of recurrence and debilitating pain, requiring long-term treatment.^[6]^ Dienogest is a form of progestin that is highly selective for progesterone receptors and acts like progesterone. Dienogest reduces the effect of estrogen on the endometrial tissue and relieves pelvic pain associated with EM.^[7]^ Dienogest (2 mg/d) has been used to control the first symptoms, and post-surgical recurrence of EM, and its efficacy and safety have been demonstrated in many clinical studies.^[6–8]^

Common adverse reactions of dienogest include headache, breast discomfort, uterine bleeding, acne, and weight gain.^[3,7,9]^ Strowitzki et al^[3]^ performed a pooled analysis of 4 randomized, controlled studies conducted in Europe comprising 332 patients treated with dienogest (2 mg once daily). Depressed mood was reported in 5.1% of the women as one of the most common adverse drug reactions. All the adverse reactions were generally mild to moderate in intensity. In a retrospective practice-based study that assessed the efficacy and safety of dienogest in 37 women with EM treated for at least 60 months, 4 reported occasional phases of depressed mood.^[5]^ However, psychiatric consultation was not required as the symptoms of depression improved following estrogen treatment. Moehner et al^[2]^ reported a slightly increased risk of depression among dienogest users in a large, prospective, surveillance study: the adjusted hazard ratios were 1.8 (95% confidence intervals, 0.3–9.4) for dienogest versus other approved medications for EM and 1.5 (95% confidence intervals, 0.8–2.8) for dienogest versus hormonal treatments not approved but frequently used for EM treatment. They concluded that this increased risk might result from a potentially more severe form of EM in dienogest users at baseline or unknown country-specific confounding variables, not excluding the possibility of an actual increased risk of depression for dienogest. In most studies, the time interval for the occurrence of depression associated with dienogest use was not described in detail.

Given the previous reports that dienogest could be related to the occurrence of mild to moderate depression, and the relationship might be explained by other confounding factors, it seems unusual that the patient in this report experienced a severe episode of depression with suicidal thoughts after taking dienogest (2 mg/d) for 2 months. She was promptly referred for psychiatric consultation. This was different from the other cases seen in the analysis of the long-term treatment of EM with dienogest, in which women developed phases of depressed mood but did not require psychiatric consultation.^[5]^ Although severe symptoms of MDD, including anergia, rage, crying spells, and suicidal thoughts, had improved on the second visit to the psychiatric outpatient clinic, 2 weeks after discontinuation of dienogest, other symptoms, such as depressed mood, anxiety, and insomnia continued; moreover, the patient was under close observation with an antidepressant medication. She was in a state of remission for the major depressive episode at the 16 weeks of psychiatric consultation and was on maintenance therapy for 9 months. Subsequently, the psychiatrist recommended discontinuation of the antidepressant medication. Strowitzki et al^[10]^ reported a case of 1 woman who experienced severe depression during a 24-week, randomized, multicenter study with dienogest (2 mg/d) in 124 subjects, and the importance of close monitoring for a depression history in these patients was emphasized. However, the patient in this case, did not have a history of depressive disorder or other psychiatric disorders. Given the abrupt onset of a major depressive episode after taking dienogest and a significant improvement of its severe symptoms after discontinuation of the medicine, dienogest was considered as the more important factor in the development of depression than any others such as the surgery and EM. Although there are alternative medicines, such as monophasic estrogen–progestin and depo–provera for the treatment of EM approved by the U.S. Food and Drug Administration,^[11]^ the gynecologists did not recommended further hormonal treatment for the patient.

The brain is an important target for sex hormones. Receptors for sex hormones, including progesterone A and B, are highly expressed in various areas and have specific effects on neuromodulation. These hormones might act on the receptors in regions of the brain that are crucial for cognitive and emotional processing. These include the hypothalamus, amygdala, and prefrontal cortex.^[12,13]^ Progestins such as dienogest, which act like a progesterone, have been known to change the levels of progesterone and its metabolite, allopregnanolone, in the plasma and different regions of the brain.^[14,15]^ These altered levels can affect the activity of the γ-aminobutyric acid (GABA)-A receptors by inducing γ^2^ subunit gene expression, which has been associated with the development of anxiety and depression.^[16]^ However, these changes in neurosteroid levels and GABA responses do not always seem to be related to the risk of depression in progestin users. Rapkin et al^[17]^ reported no association between decreased neurosteroids levels and adverse mood changes, and Huber et al^[18]^ suggested a positive effect of progestins on depressed mood. Therefore, it has been suggested that the individual vulnerability of the brain to hormonal fluctuations might affect the occurrence of depressive disorders. Some subgroups of women prone to mood disorders related to hormonal effects have been suggested in previous studies. Adolescents seem to be more predisposed to develop a negative mood during the intake of female hormonal preparations.^[19,20]^ Hamstra et al^[21]^ reported that female carriers of mineralocorticoid receptor haplotype 1 or 3 showed sensitive responses towards sad and fearful faces and emotional memory during the use of oral contraceptives. Those with a previous history of mental disorders had a greater risk of adverse mood symptoms induced by combined oral contraceptives,^[22]^ and progestins have been shown to have the potential to develop negative moods in women with or without baseline depressive disorders.^[23]^

Clinicians should take a careful and accurate history of mood disorders and evaluate the factors that can lead to depression before prescribing hormonal preparations, especially progestins. Nevertheless, it is noteworthy that severe depressive disorder with suicidal ideation can occur in women taking progestins despite no previous history of psychiatric disorders and no presence of risk factors, as in this case. In addition, this report highlights the importance of patient education before and during therapy and close observation of mood changes by clinicians. If a negative mood appears, prompt psychiatric consultation should be recommended to prevent progression to a state of a more severe depressive disorder.

## Author contributions

**Conceptualization:** Jin Kyung Park.

**Data curation:** Sang Min Lee, Jin Kyung Park.

**Writing – original draft:** Sang Min Lee, Jin Kyung Park.

**Writing – review & editing:** Jin Kyung Park.
